# From Molecules to Biomarkers: Nogo Proteins and Receptors in the Early Detection of Type 2 Diabetes Complications: A Systematic Review

**DOI:** 10.3390/ijms27115124

**Published:** 2026-06-05

**Authors:** Jelena M. Bogdanović, Ivana Babić, Jelena Stanarčić Gajović, Sandra Singh Lukač, Dragana Mijač, Dušan Popović, Ivan Ranković, Ljiljana Popović, Iva Rasulić, Katarina Lalić

**Affiliations:** 1 Clinic for Endocrinology, Diabetes and Metabolic Diseases, University Clinical Center of Serbia, 11000 Belgrade, Serbia; 2 School of Medicine, University of Belgrade, 11000 Belgrade, Serbia; 3 Emergency Center, University Clinical Center of Serbia, 11000 Beograd, Serbia; 4 Clinic for Gastroenterology and Hepatology, University Clinical Center of Serbia, 11000 Beograd, Serbia; 5 Department for Gastroenterology and Hepatology, University Hospital Center Dr Dragiša Mišović-Dedinje, 11000 Beograd, Serbia

**Keywords:** type 2 diabetes, Nogo/RTN4, biomarkers, RhoA/ROCK, GLP-1

## Abstract

Nogo (RTN4) proteins and their receptors have emerged as candidate mediators of metabolic regulation and vascular pathology relevant to type 2 diabetes (T2D). The primary objective of this PRISMA-guided systematic review was to evaluate the clinical and cohort evidence for RTN4/RTN4R as potential biomarkers of T2D progression and vascular complications. A secondary objective was to synthesize preclinical mechanistic evidence on the effects of Nogo axis modulation on pathways relevant to the pathogenesis of T2D. We performed a PRISMA-guided systematic review. The protocol was not prospectively registered in PROSPERO. To ensure reproducibility, we provide complete search keywords, the screening log and the full-text exclusion table. PubMed/MEDLINE, EMBASE and Web of Science were searched for studies published 2000–2025; full search keywords are provided in the main text. The search strategy combined and free-text terms with Boolean operators. We included original preclinical and clinical studies, cohort/proteomic analyses, meta-analyses, and mechanistic papers reporting expression, function, signaling, or clinical associations of Nogo proteins/receptors in metabolic or vascular outcomes. Exclusion criteria: non-English articles, unclear methods, studies outside 2000–2025, and studies lacking primary data. Two reviewers independently screened records; conflicts were resolved by consensus. Study quality was appraised using established tools (SYRCLE for animal studies, Newcastle–Ottawa Scale for cohort/case-control studies). Preclinical evidence supports tissue-specific roles for RTN4 isoforms and receptors in the regulation of insulin secretion, proGCG → GLP-1 processing, ER homeostasis, and vascular permeability through the Src/PI3K/Akt and RhoA/ROCK axes. Cohort and proteomic analyses report associations between RTN4/RTN4R or serum NogoB and faster progression of T2D or vascular complications, but genetic assessment of causality (Mendelian randomization) has so far provided limited support in available data sets. Findings are heterogeneous with respect to directionality and tissue localization. RTN4 signaling exhibits tissue-specific mechanisms relevant to glucose regulation and vascular biology and warrants further translational study. However, heterogeneity across studies and limited genetic support for causality indicate that isoform-specific quantitative validation, longitudinal cohorts and integrated genetic–functional analyses are required before RTN4/RTN4R can be considered as clinical biomarkers.

## 1. Introduction

Diabetes mellitus (DM) is one of the most widespread chronic metabolic disorders, with an increasing incidence globally. It is characterized by elevated blood glucose levels due to insulin deficiency (type 1) or insulin resistance (type 2). Despite advances in the diagnosis and treatment of DM, early detection remains challenging, and current biomarkers—such as fasting blood glucose, HbA1c, and insulin levels—have limitations in sensitivity and specificity. Furthermore, these markers do not adequately reflect the neural and vascular complications that may be associated with DM [1,2]. Due to the slow development of type 2 DM (T2D), it often remains undiagnosed for a long time and is frequently discovered by chance, often already at the stage when chronic complications of DM have developed. For these reasons, early detection and proactive monitoring are key to preventing complications and improving patients’ quality of life. Consequently, research into new clinically applicable biological indicators that reflect pathophysiological processes is essential.

The prevalence of T2D has increased markedly worldwide over recent decades. Reports from the International Diabetes Federation (IDF) indicate a rise from approximately 425 million adults with DM in 2017 to an estimated 589 million in 2024, with projections of further increases in coming decades [1,3,4]. Prevalence varies by region and income level (e.g., 12.5% in high-income vs. 6.1% in low-income countries in 2024) and increases with age. However, rising obesity and sedentary lifestyles have also driven earlier onset in younger populations [1,5,6]. DM is a major cause of morbidity and mortality worldwide, leading to microvascular complications (retinopathy, nephropathy, neuropathy) and macrovascular disease (increased risk of cardiovascular events and stroke) [5].

The pathogenesis of T2D is multifactorial, involving genetic predisposition, lifestyle, and environmental factors that converge on insulin resistance and progressive β-cell dysfunction [7,8,9,10,11,12]. Insulin resistance—particularly in skeletal muscle, adipose tissue, and liver—results from impaired insulin signaling, reduced peripheral glucose uptake, and increased hepatic glucose production. Adipose tissue-derived adipokines and proinflammatory cytokines (e.g., TNF-α, IL-6) further disrupt signaling and exacerbate resistance [13,14,15]. Initially, pancreatic β-cells compensate by increasing insulin secretion, but chronic glucotoxicity, lipotoxicity, oxidative and endoplasmic reticulum (ER) stress lead to β-cell dysfunction, loss of mass, and apoptosis, driving the transition to overt hyperglycemia [16,17,18,19].

Insulin action in the liver critically involves the transcription factor FOXO1. In health, insulin signaling suppresses FOXO1 activity, thereby inhibiting gluconeogenic gene expression. In insulin resistance, impaired suppression of FOXO1 leads to increased expression of gluconeogenic enzymes and heightened hepatic glucose output, contributing substantially to fasting hyperglycemia and overall glycemic dysregulation [20,21,22,23]. Concurrent ER stress, inflammation, and lipid disturbances further aggravate β-cell dysfunction and systemic insulin resistance [24,25].

Additional contributors to T2D pathogenesis include incretin dysfunction involving glucagon-like peptide-1 (GLP-1) and glucose-dependent insulinotropic polypeptide (GIP), chronic inflammation, oxidative stress, gut microbiome alterations, and epigenetic changes, all of which shape disease onset and progression [26,27].

Recent research has highlighted complex neurovascular interactions in metabolic regulation: the nervous system (central and peripheral) plays vital roles in energy balance, appetite control, and insulin sensitivity [28,29,30,31]. Nogo proteins (RTN4 isoforms) and their receptors (NogoR/RTN4R), originally characterized for inhibition of axonal regeneration in neurobiology [32], have emerged as regulators of peripheral metabolic and vascular processes and potential biomarkers/modulators of T2D pathogenesis [33,34,35].

**Biological role of Nogo proteins and receptors:** Nogo proteins are an important group of molecules in neurobiology whose primary role is the inhibition of nerve-fiber regeneration in the central nervous system (CNS) [36,37]. They belong to the reticulon family and are expressed predominantly in CNS myelin sheaths. Encoded by the reticulon 4 (RTN4) gene (NOGO), three main isoforms are described in humans and experimental animals: NogoA, NogoB, and NogoC [36,38]. These isoforms share a C-terminal reticulon homology domain of ~188 amino acids containing two hydrophobic transmembrane regions separated by a functional 66-AA loop (Nogo-66) responsible for axon-growth inhibition [36,38].

NogoA, the most studied isoform, is a 200 kDa protein that contains an additional amino-Nogo domain localized to the endoplasmic reticulum and is mainly expressed in oligodendrocytes. Its expression increases after injury and limits regeneration, while NogoA removal improves axonal regrowth in experimental models [37,38,39,40]. NogoB (~25 kDa) is abundant in muscle tissue; skeletal muscle dedifferentiation alters the balance of these isoforms (↑ NogoA, ↓ NogoC) [36,41,42].

**Scheme 1:**
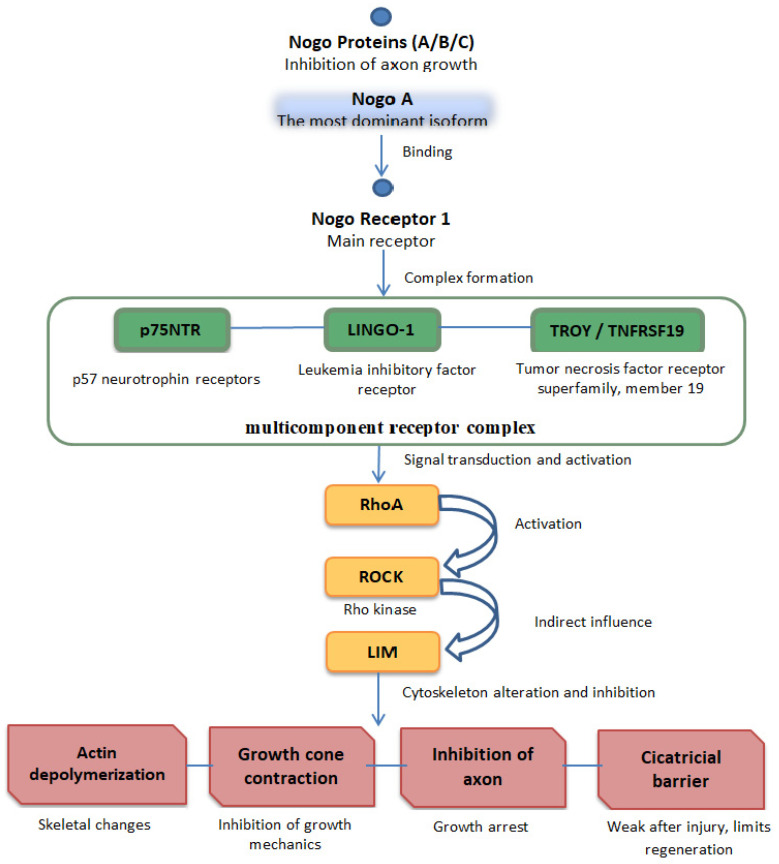
Representation of the RhoA/ROCK signaling pathway.

**Molecular structure and signaling pathways of Nogo proteins and receptors:** Beyond CNS regeneration, Nogo proteins and receptors act in peripheral tissues and regulate metabolic processes, including insulin secretion, glucose uptake, adipogenesis, and inflammation [36,43,44]. Nogo isoforms function via multicomponent receptor complexes rather than individually. Nogo receptors are GPI (glycosylphosphatidylinositol)-anchored leucine-rich repeat proteins (NogoR1/2/3) that require coreceptors to transduce signals [36]. GPI anchoring occurs during post-translational processing in the ER, leaving NogoRs without cytoplasmic domains and dependent on coreceptors (p75NTR (p75 neurotrophin receptor), TROY/TNFRSF19 (tumor necrosis factor receptor superfamily member 19), and LINGO1 (leucine-rich repeat and Ig domain-containing Nogo receptor-interacting protein 1)) for intracellular signaling [36,45,46,47]. NogoR1 recognizes NogoA/Nogo-66 and other myelin-associated inhibitors (MAG); NogoR2 binds MAG specifically, while NogoR3 does not interact with MAG [36,40,45]. Ligand binding (e.g., NogoA → NgR1) induces oligomerization with coreceptors and activates intracellular cascades centered on the RhoA–ROCK axis. RhoA activation stimulates ROCK, which activates LIM kinase (LIMK). LIMK phosphorylates and inactivates cofilin, reducing actin turnover, while ROCK increases myosin-mediated contractility. These changes disrupt actin/microtubule dynamics in the growth cone, causing growth-cone collapse and inhibition of axonal extension (see Scheme 1) [36,46,47,48].

**Nogo proteins and receptors—potential mechanisms in DM:** Experimental and translational data indicate tissue-specific effects on glucose homeostasis and complications: (a) modulation of insulin granule mobilization and exocytosis in β-cells (NogoA) [43,49,50,51,52,53,54]; (b) interference with prohormone processing in enterocytes, reducing active GLP-1 (NogoB–proGCG/PCSK1 axis) [35]; (c) influence on ER membrane homeostasis, dolichol-dependent glycosylation, and hepatic Akt signaling via the NogoB receptor (NGBR/RTN4BR) [24,44,55,56]; and (d) activation of endothelial signaling cascades (Src → PI3K/Akt/ERK and RhoA → ROCK) that increase vascular permeability and promote neurovascular injury relevant to diabetic complications [57,58]. Proteomic and cohort studies further support translational relevance (RTN4/RTN4R associations with faster T2D progression and associations between serum NogoB levels and vascular complications), but quantitative validation, standardized assays, and prospective causal studies are required before clinical implementation as reliable biomarkers [33,59].

Given the epidemiological burden of T2D, the limitations of existing biomarkers, and the mechanistic evidence implicating Nogo proteins and receptors across the pancreas, gut, liver, and vasculature, the RTN4 system represents a promising translational target.

The primary objective of this PRISMA-guided systematic review was to evaluate the clinical and cohort evidence for RTN4/RTN4R as potential biomarkers of T2D progression and vascular complications. A secondary objective was to synthesize preclinical mechanistic evidence on the effects of Nogo axis modulation on pathways relevant to the pathogenesis of T2D. The results are presented separately for human/clinical evidence and mechanistic/preclinical evidence.

## 2. Methodology

### 2.1. Protocol and Registration

This review was conducted according to the PRISMA 2020 statement. PRISMA offers a consistent and replicable method for identifying relevant literature. It also provides guidance for selecting, screening, and assessing research articles. The review protocol was not prospectively registered in PROSPERO because it had not been registered at the time of the review and no quantitative meta-analysis was planned due to substantial methodological and biological heterogeneity, quantitative analysis was not appropriate. To ensure reproducibility, we provide complete search strings (Supplementary Table S1), included studies (Supplementary Table S2), and the quality assessment table (Supplementary Table S3).

Details of the process are presented in the following subsections.

### 2.2. Information Sources and Search Strategy

Three electronic databases were used to conduct the search procedure and collect relevant papers. The databases searched in this study included PubMed/MEDLINE, EMBASE, and Web of Science for publications between 2000 and 2025. We selected the period from 1 January 2000, to 31 December 2025, to capture early molecular and neurobiological studies of RTN4/Nogo family members while also including work produced using modern technologies (mass spectrometry, proteomics, targeted ELISA, and genetic analyses) that became widely available during the 2000s. The start date of 2000 enabled coverage of the early literature, while the upper bound of 31 December 2025 was selected to include the most recent translational and multi-omics studies—particularly studies published from approximately 2013 onward that first linked Nogo biology to metabolic processes—and to ensure measurement consistency and biomarker relevance. Search strategies combined controlled vocabulary (MeSH in PubMed and EMTREE in EMBASE) with free-text terms using Boolean operators and truncation where appropriate.

*Keywords for PubMed/MEDLINE:* (“Reticulon 4”[MeSH] OR “Reticulon-4”[tiab] OR “RTN4”[tiab] OR “Nogo”[tiab] OR “NogoB”[tiab] OR “NogoR”[tiab] OR “RTN4R”[tiab]) AND (“Diabetes Mellitus, Type 2”[MeSH] OR “type 2 diabetes”[tiab] OR “T2D”[tiab] OR “diabetes”[tiab]) AND (“Biological Markers”[MeSH] OR “biomarker*”[tiab] OR “proteom*”[tiab] OR “GLP-1”[tiab] OR “proGCG”[tiab] OR “incretin*”[tiab] OR “insulin”[tiab] OR “retinopathy”[tiab] OR “nephropath*”[tiab]).

*Keywords for EMBASE (Elsevier)*: (reticulon OR rtn4 OR nogo) AND (type 2 diabet OR t2d) AND (biomarker OR proteom OR incretin OR cohort).

*Keywords for Web of Science:* (reticulon* OR RTN4 OR “Reticulon 4” OR nogo* OR RTN4R) AND (“type 2 diabet*” OR T2D OR “diabetes mellitus type 2” OR diabetes) AND (biomarker* OR proteom* OR “GLP-1” OR proGCG OR incretin* OR insulin OR retinopath* OR nephropath*).

Complete database-specific search keywords are provided in Supplementary Table S1. The last search date was 30 April 2026. In all databases we applied publication-year filters. Therefore, manuscripts from 1 January 2000 to 31 December 2025 were included.

**Figure 1:**
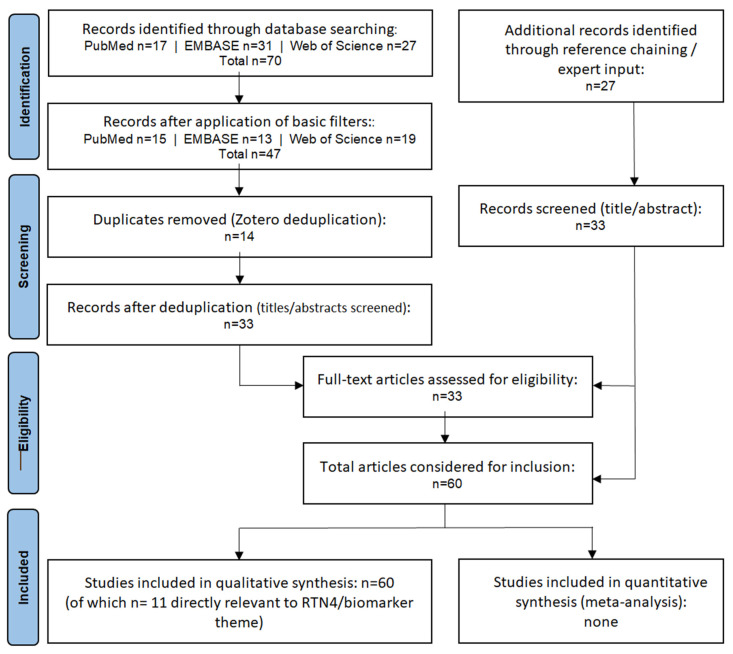
PRISMA flow diagram.

Initially, 75 database records were identified (PubMed 17; EMBASE 31; Web of Science 27). After applying language and basic inclusion criteria, 47 unique records remained (PubMed 15; EMBASE 13; Web of Science 19). All records were imported into Zotero; automated and manual deduplication removed 14 duplicates, leaving 33 records for title and abstract screening. In addition to the database search, reference chaining of included articles and screening of relevant review articles were conducted, leading to the identification of additional relevant papers. In total, 60 references were included in the analysis; of these, 11 studies were identified as directly relevant to the main topic and presented in detail in tabular form, while the remaining references were used for interpretation, context, and mechanistic descriptions (see Figure 1 for the PRISMA flow diagram).

### 2.3. Eligibility Criteria

Inclusion criteria: (1) original human clinical studies (cohort, cross-sectional, and case-control studies), proteomic analyses, or preclinical mechanistic studies reporting expression, functional data, or clinical associations of Nogo proteins/receptors in type 2 diabetes or its complications (including in vitro experiments, animal models, proteomics, ELISA, Western blot, immunohistochemistry, and functional molecular studies); (2) original research articles or reviews containing primary data; (3) studies published in English; and (4) studies published between 2000 and 2025.

Exclusion criteria: (a) studies that did not present original numerical data (e.g., comments or opinion papers only); (b) studies without a sufficient description of the measurement methods used to allow replication of the main procedures; (c) preclinical studies with n < 3 in experimental groups for key outcomes or without descriptions of randomization/blinding for outcomes subject to bias; and (d) studies published outside the search period (before 2000 or after 31 December 2025) or without full-text availability in English.

### 2.4. Study Selection and Data Extraction

Two reviewers (JB, IB) independently screened titles, abstracts, and full texts and extracted data into a standardized Excel data collection form, including author, publication year, study type/model, sample size (n), sample/tissue type, method/assay, main numerical outcomes (where available), and reason for inclusion (mechanistic, biomarker, or translational relevance). Data extraction forms and study summaries were retained as Supplementary Material and linked to the corresponding source figures and tables (Supplementary Table S2 and Table 1). Disagreements were resolved through discussion and consensus.

### 2.5. Risk of Bias and Quality Assessment

Study quality was appraised using standardized tools for both human observational and preclinical studies. Human studies were evaluated using the Newcastle–Ottawa Scale (NOS), whereas preclinical studies were assessed using SYRCLE’s risk-of-bias tool (available at https://www.syrcle.nl/, accessed 10 May 2026 and 11 May 2026) Assessments were performed independently by two reviewers (JB, IB). Each domain was evaluated and documented. Disagreements were resolved by consensus. Detailed risk-of-bias assessment tables are provided in Supplementary Table S3.

### 2.6. Data Synthesis

Results were synthesized in two streams: (A) human/clinical evidence (cohort studies, proteomics, and serology) addressing biomarker potential; and (B) mechanistic/preclinical evidence (in vitro and in vivo studies) addressing causality and molecular pathways. Due to the heterogeneity of study designs, outcome measures, and measurement methods, a narrative synthesis integrating preclinical and clinical findings was conducted. No quantitative meta-analysis was performed.

## 3. Results

During the initial search, 75 records from different databases were identified. After applying language and eligibility criteria, 47 unique records remained. These records were then imported into Zotero (7.0.30 (64-bit)), where 14 duplicate records were automatically and manually removed, resulting in 33 remaining papers for title and abstract screening. Through additional searches of references and relevant review articles, further studies were identified. In total, 60 references were included in the analysis, of which 11 were directly relevant and listed in Table 1, while the remaining studies were used for contextual interpretation and discussion (see Figure 1, PRISMA flow diagram). After full-text review, certain papers were excluded because they did not meet the inclusion criteria: studies irrelevant to RTN4 in the context of diabetes, commentaries and editorial reviews without original data, and abstracts without available full text (unless they provided unique data). While other references were used for interpretation, context, and mechanistic description, the omitted papers did not contribute directly to evidence relevant to the research questions. The characteristics of the included studies are presented in Table 1 and Supplementary Table S2.

### 3.1. Integrative Conclusions of the Qualitative Assessment: SYRCLE (Animal Studies) and NOS (Cohort Studies)

A comprehensive analysis points to frequent methodological flaws in preclinical animal research according to SYRCLE criteria. Frequent deficiencies or ambiguities in the documentation of randomization, allocation concealment, and blinding result in a high or unclear risk of bias in domains such as selection, performance, and detection biases. In addition, small sample sizes and, in some cases, short treatment duration reduce the reliability of results, especially for subjective outcomes such as histology and image quantification. In contrast, NOS-rated cohort studies demonstrate high methodological quality, with large, well-characterized cohorts, robust biomarker measurement, adjustments for key confounders, and replication, contributing to the credibility of associations. The combination of evidence from both categories suggests that the synergy between high-quality cohort data and functional preclinical research can strengthen translational hypotheses.

### 3.2. Preclinical Studies

#### 3.2.1. Pancreas—β-Cell Expression and Insulin Secretion

A 2013 study by Bonal et al. [43] showed in experimental models that reduction in NogoA significantly improves insulin secretion. Bonal et al. investigated the role of reduced NogoA expression in β-cell function of the islets of Langerhans and regulation of insulin secretion. Methods included RT-PCR and Western blot for RTN4A/NogoA in isolated islets and Pdx1-EGFP β-cells, immunohistochemical staining for visualization in islets, in vivo glycemic tolerance tests, insulin tolerance tests and vagal stimulation assays, and in vitro GSIS (glucose-stimulated insulin secretion) in isolated islets. The number of samples varied by study (islet assays n = 3–15; in vivo assays listed individually).

In the first part of the experiment, C57BL/6J NogoA knockout (KO) male mice and age-matched wild-type (WT) controls were used. RTN4A/NogoA expression was confirmed in isolated islets and Pdx1-EGFP β-cells (RT-PCR, Western blot). Structural and phenotypic outcomes were evaluated by immunohistochemical staining, in vivo metabolic tests (ipGTT 2 g/kg after an overnight fast; ivGTT 1 g/kg with/without 0.53 mmol/L carbachol; insulin tolerance test 0.5 U/kg), vagal stimulation (2-DG, 50 mg/kg i.v.), as well as in vitro GSIS on isolated islets (10 islets/batch; glucose 1.4–16.8 mmol/L ± carbachol 10 mmol/L or GLP-1 100 nmol/L). The amounts of pancreatic insulin and glucagon were quantified by extraction in acidic ethanol, and the amount of insulin-positive tissue (β-cells) was assessed by immunohistochemical staining for insulin (quantification of insulin-positive surface or volume, converted to mass). Compared to WT, KO mice in the non-fasted state had lower glucose levels (WT 10.66 ± 0.35 mmol/L vs. KO 8.48 ± 0.31 mmol/L, n = 5, *p* = 0.04) and higher plasma insulin levels (WT 0.50 ± 0.02 mg/L vs. KO 1.23 ± 0.28 mg/L, n = 6, *p* = 0.04), while glucagon remained unchanged. During ipGTT, cumulative glycemia (AUC 0–120 min) was significantly lower in KO mice (WT 709.88 ± 72.73 vs. KO 378.95 ± 31.17 mmol/L·min, n = 4, *p* = 0.01), with increased stimulated plasma insulin levels in KO mice. Upon simultaneous administration of carbachol and glucose, KO mice had increased insulin secretion compared to the control group. Isolated KO islets showed higher insulin secretion under the combination of glucose and carbachol (e.g., 1.4 mmol/L + CC: WT 0.070 ± 0.008% vs. KO 0.195 ± 0.025%, *p* < 0.005; 8.4 mmol/L + CC: WT 0.170 ± 0.025% vs. KO 0.287 ± 0.036%, *p* < 0.005; n = 7–13, *p* < 0.05), while responses to glucose alone and to glucose + GLP-1 were similar between groups. β-cell mass, islet architecture, and pancreatic glucagon content were not increased. In KO mice, the amount of insulin in the pancreas was moderately higher (WT 554.65 ± 54.54 ng vs. KO 763.51 ± 37.67 ng; n = 5; *p* < 0.05).

In the second part of the experiment, in the db/db model of early T2D, animals (5 weeks old) received two i.v. injections of a neutralizing anti-NogoA monoclonal antibody (called 11C7) (4.9 mg total per mouse for 2 weeks) or a control antibody. Pharmacological neutralization was associated with higher plasma insulin levels (control 13.94 ± 5.02 mg/L vs. 11C7 32.28 ± 8.14 mg/L, n = 3; *p* < 0.05) and higher stimulated insulin levels during ipGTT (15 min: control 1.25 ± 0.08 mg/L vs. 11C7 2.17 ± 0.15 mg/L, n = 3–4; *p* < 0.05). Following intravenous glucose administration with carbachol, insulin secretion was significantly enhanced in the treated group (control 10.65 ± 2.14 vs. 11C7 46.43 ± 8.00, n = 3; *p* < 0.05).

#### 3.2.2. Intestinal/Enteroendocrine Regulation of GLP-1

Gong et al. [35] investigated the role of NogoB using Nogo−/− mice and intestinal-specific Nogof/f;VillinCre lines, STC-1 enteroendocrine cell experiments, HEK293T co-IP/MS analyses, ELISA and SPR binding assays, AAV and siRNA manipulations, and several experimental models of diabetes (HGD and STZ/HFD). The study additionally analyzed human duodenal samples from individuals with and without T2D (obtained from previously prepared paraffin blocks).

In quantitative in vitro assays, purified human NogoB showed binding to human proGCG in ELISA (Kd ≈ 21.17 µg/mL) and SPR assay (K D ≈ 1.374 µM). Overexpression of NogoB in STC-1 cells led to retention of proGCG in the ER, increased intracellular levels of proGCG, and decreased secretion of GLP-1, while knockdown of NogoB decreased levels of proGCG and PCSK1 and increased secreted GLP-1. In knockout mouse models (Nogof/f;VillinCre) with intestinal-specific absence of NogoB, especially under a high-glycemic diet or in STZ/HFD models of diabetes, increased concentrations of circulating active GLP-1 and insulin, decreased glucagon, improved glucose tolerance and GSIS, decreased activity of hepatic gluconeogenic enzymes, increased expression of GLP-1 receptor and insulin gene in the pancreas, increased proportion of β-cells, reduced proportion of α-cells, and reduced islet apoptosis were observed. Immunofluorescence analysis showed an increased number of GLP-1-positive cells and enhanced colocalization of proGCG and PCSK1 in the intestine. In human duodenal samples, the authors showed stronger NogoB expression in samples from individuals with T2D, along with reduced colocalization of PCSK1 and proGCG.

#### 3.2.3. Liver Metabolism and NGBR

Chen et al. [44] investigated the impact of hepatic RTN4BR expression using hepatocyte-specific RTN4BR knockout mice (NGBRhepKO) and AAV9-mediated overexpression of RTN4BR in HFD/STZ-induced T2D models, with complementary in vitro experiments in HepG2 cells, primary hepatocytes, and the AMPKα1-knockout HepG2 line. The methodology included systemic administration of AAV9-NGBR (1 × 10^12^ vg/mouse), metabolic phenotyping (GTT, ITT, hyperinsulinemic-euglycemic clamp), liver histology (Oil Red O, Nile Red), measurements of serum lipids and liver enzymes (TG, NEFA, AST, ALT, and TBIL), quantification of ER-stress and insulin pathway markers (Western blot for p-AKT, p-GSK3β, p-AMPK, p-INSR/IRS; BIP, PERK, p-eIF2α), and cell manipulations with siRNA and overexpression.

In NGBRhepKO animals, an approximately 40% increase in fasting glucose compared with controls, pronounced insulin resistance in GTT/ITT, development of liver steatosis, and increased proinflammatory factors (TNFα, IL-1β) were recorded. In addition, decreased levels of plasma insulin and C-peptide were recorded, along with a decrease in the mass of insulin-positive β-cells. Experimental upregulation of RTN4BR expression by AAV9 was associated with decreased hepatic triglycerides; improved liver histopathology, reflected by less lipid accumulation on Oil Red O staining; reduced levels of ALT/AST and total bilirubin in serum; better glucose control and lower insulin levels; and reduced apoptotic activity of β-cells, as demonstrated by the TUNEL method.

Biochemical analyses point to a mechanism in which RTN4BR plays a key role in regulating insulin signaling and cellular stress. Increased expression of RTN4BR leads to increased phosphorylation of AKT and GSK3β in the liver and muscle cells, as well as activation of AMPK (p-AMPK), with a decrease in ER-stress markers (BIP, PERK, p-eIF2α, ATF4, XBP1s). After RTN4BR suppression, increased ER stress and decreased insulin signaling were recorded. Experiments performed in cell models showed that the effects of RTN4BR on insulin signaling are dependent on AMPKα1, since knockout of AMPKα1 prevents restoration of RTN4BR-mediated insulin signaling.

#### 3.2.4. Retina—RTN4R and ROCK; RGC Apoptosis

Liu XZ et al. [57] conducted an experimental study on 40 adult Sprague–Dawley rats randomly assigned to four groups: control, diabetes, intravitreal administration of siRTN4R, and siRNA blank (n = 10 per group). Diabetes was induced with streptozotocin (STZ 60 mg/kg i.p.), intravitreal injection of lentivirus (10 µL) carrying siRTN4R or siRNA blank was performed after induction of diabetes, and outcomes were assessed after 12 weeks. The methods included double fluorescent staining for RTN4R and the retinal ganglion cell marker Brn3a, immunohistochemistry and Western blot for ROCK, TUNEL assay for RGC apoptosis, and quantification in four randomly selected fields per retina (each field read in triplicate).

Western blot and immunohistochemical analyses showed upregulation of RTN4R and ROCK in the retina in the diabetic group and the control siRNA group compared with the control group (*p* < 0.01). In the group treated with siRTN4R, the levels of RTN4R and ROCK were not statistically different from those in the control group (*p* > 0.05). The proportion of ROCK-positive retinal ganglion cells (RGCs) was 9.1 ± 0.5% in the control group, 35.2 ± 1.7% in the diabetes group, and 30.6 ± 2.1% in the siRNA blank group (*p* < 0.01 for comparison with control). In the siRTN4R group, the proportion was 11.6 ± 0.7% (*p* < 0.05 vs. diabetes). Assessment of apoptosis using the TUNEL method showed a low level of TUNEL positivity in the control group, while the diabetes and siRNA blank groups showed high proportions of TUNEL + RGCs (46.8 ± 3.5% and 51.3 ± 2.1%); in the siNgR group, the proportion of TUNEL + RGCs was 18.7 ± 1.5% (*p* < 0.01 vs. diabetes).

#### 3.2.5. Vascular/Endothelial Function—HUVEC and BRB

Yang Q et al. [58] (with supporting data from Irakoze et al. [59]) examined RTN4/NogoB in retinal vascular permeability and endothelial function using STZ-induced diabetic rat retina with intravitreal AAV-siNogoB, ELISA measurements of human plasma and vitreous samples, and in vitro HUVEC experiments with high glucose (25 mmol/L) ± cholesterol (100 µM) combined with lentiviral NogoB overexpression or knockdown. Retinal barrier integrity was assessed by FITC/Rhodamine dextran leakage, junctional proteins (ZO-1, occludin, VE-cadherin) by Western blot/qPCR and immunostaining, and signaling by phosphorylation assays for Src, Akt, and ERK. HUVEC assays included tube formation, wound healing, cytokine ELISAs, and markers of endothelial-to-mesenchymal transition.

In human samples, plasma NogoB concentrations were higher in patients with diabetes and edematous/microvascular complications and in proliferative diabetic retinopathy compared with non-diabetic controls (approximately 3.3- and 6.0-fold increases, respectively), whereas vitreous NogoB was not altered. In diabetic rats, retinal vascular leakage was markedly increased (≈15.7-fold at 4 weeks), and intravitreal NogoB knockdown reduced leakage by ≈26.6% (*p* = 0.007), while partially restoring ZO-1 and VE-cadherin expression toward control levels. Diabetic retina exhibited altered signaling (reduced inactive p/t Src [Tyr529], increased p/t Akt, and increased p/t ERK), changes that were modulated toward control values by NogoB knockdown. In HUVECs, high glucose ± cholesterol increased proinflammatory cytokines (IL-1, IL-6, TNF-α, TGF-β1), decreased IL-10, and reduced viability, tube formation, and wound closure. NogoB knockdown exacerbated these dysfunctions, whereas NogoB overexpression improved endothelial function and reversed endothelial-to-mesenchymal transition (EndMT) markers. These data describe context-dependent roles for NogoB in vascular biology: physiological NogoB appears necessary for endothelial/junctional homeostasis in the normal retina, while diabetes-associated dysregulation of NogoB is associated with blood–retinal barrier (BRB) breakdown and proinflammatory/EndMT responses via Src/Akt/ERK and junctional protein perturbation.

#### 3.2.6. Renal Protection by Circulating sNogoB

Hernandez Diaz et al. [60] examined the effect of elevated levels of the secretory fragment NogoB (sNogoB) in an STZ-induced model of diabetes using DBA/2J mice treated with an AAV/DJ vector expressing 6 × His-tagged sNogoB (AAV-sNogoB) compared to AAV-GFP, with follow-up for 12–14 weeks. After injection of AAV-sNogoB, an approximately 12-fold higher concentration of circulating sNogoB was measured. The methods included quantification of circulating and urinary sNogoB by ELISA, 24 h albuminuria and creatinine clearance, electron microscopy and lectin staining to assess the glomerular glycocalyx, Western blot/ELISA analysis of VEGF-A, Angpt1/2 and phosphorylated VEGFR2, and measurements of p-eNOS, p-AKT, p-GSK3β, and β-catenin, with IP/PLA experiments to detect the sNogoB–RTN4BR interaction in glomerular endothelial cells (GECs).

Compared to control diabetic animals, increased circulating sNogoB was associated with a reduction in diabetic albuminuria by about 40–50% (D-GFP vs. D-sNogoB, *p* = 0.04). Lectin staining showed that glycocalyx thickness was reduced in diabetes, while increased or overexpressed sNogoB restored the lectin signal to levels comparable to control (*p* values ≤ 0.009 to 0.0004 for the indicated comparisons). Diabetes increased VEGF-A expression and VEGFR2 phosphorylation, changes that were reduced by increased or overexpressed sNogoB. At the same time, increased levels of sNogoB led to normalization of p-eNOS and p-AKT, as well as a decrease in p-GSK3β(Ser9) and an increase in β-catenin signaling in diabetes. In vitro IP/PLA experiments indicated a direct interaction between sNogoB and RTN4BR in human GECs.

#### 3.2.7. Molecular Structure and Genetic Loss-of-Function

Edani et al. [56] and Park et al. [55] used a combination of structural, biochemical, and genetic methods to investigate the role of RTN4BR in cis-prenyltransferase (cis-PTase) activity and its clinical consequences. They recombinantly purified the heterodimer RTN4BR(ΔN)/DHDDS and solved its crystal structure in complex with substrates. In addition, they performed in vitro cis-PTase enzyme assays using radiolabeled IPP (isopentenyl pyrophosphate/isopentenyl diphosphate), analyzed dolichol profiles by the LC-MS method, and used yeast for complementation experiments. In the clinical part of the study, exome sequencing identified the NUS1 (RTN4BR) R290H variant in patients with a neurodevelopmental phenotype, and fibroblasts from those patients were subjected to functional tests (filipin staining for free cholesterol, cis-PTase activity, mannose incorporation, and Western blot for glycoproteins).

Structural data reveal that the C-terminal RXG motif of NgBR completes the DHDDS active site and coordinates homoallylic IPP, with the heterodimeric interface and membrane recognition elements explaining the dependence of activity on complex composition and the mechanistic effects of minor sequence changes. Experimental data confirm that the R290H variant is a loss-of-function mutation, which is why patient fibroblasts show changes in terms of free cholesterol accumulation, reduced cis-PTase activity, lower mannose incorporation, and protein hypoglycosylation (e.g., LAMP1, ICAM1). The associated clinical phenotype was severe, resembling congenital disorders of glycosylation (CDG) with neurodevelopmental manifestations.

### 3.3. Human Studies

#### 3.3.1. Cohort Multi-Omics Discovery and Validation

Slieker et al. (RHAPSODY) [34] conducted a multi-omics study of diabetes progression prognosis using three discovery cohorts (DCS, GoDARTS, ANDIS) with meta-analysis and external validation cohorts (ACCELERATE, AGES Reykjavik, MDC CC, DESIR). The outcome was time until the need for insulin therapy. The identified omics panel included 19 targeted metabolites (LC-MS), 162 lipids (after QC), and 1195 proteins measured by the SomaScan platform. Methodologically, LC-MS metabolomics, Lipotype lipidomics, and SomaScan proteomics were applied, and hazard ratios were estimated using Cox proportional hazards models in three hierarchical steps (basic: age/sex/BMI; additional: HDL and C peptide; complete: diabetes duration and glucose-lowering drugs), stratified by HbA1c, with meta-analysis and Mendelian randomization (MR) based on GWAS instruments. The most promising proteins were subjected to functional experiments in islets and in vivo.

In a meta-analysis of metabolites, higher concentrations of AADA (aminoadipic acid) and homocitrulline were associated with earlier switching to insulin (AADA HR = 1.11, 95% CI 1.01–1.22, pFDR = 0.03; homocitrulline HR = 1.12, 95% CI 1.00–1.25, pFDR = 0.04). Among lipids, certain types of short-chain triglycerides were associated with an earlier need for insulin, with the direction of this association depending on the length and degree of saturation of the acyl chains. In an assay that simultaneously measured a larger set of proteins, 98 proteins were associated with faster initiation of insulin treatment. Among the more significant were GDF15 (HR 1.34; 95% CI 1.01–1.79) and RTN4R (discovery: HR 1.33; 95% CI 1.03–1.72), with the association of RTN4R confirmed in the ANDIS cohort (HR 1.20; 95% CI 1.07–1.34).

Mendelian randomization provided limited evidence for a possible causal role of some proteins. Modest support was noted for GDF15 (β = 0.03, 95% CI 0.01–0.05, *p* = 2.68 × 10^−3^), IL-18Ra (β = 0.02, 95% CI 0.003–0.03, *p* = 0.014), and FAS (β = 0.05, 95% CI 0.005–0.09, *p* = 0.03), while evidence for metabolites and lipids was limited. Functional testing of six priority proteins (GDF15, IL-18Ra, NogoR, CRELD1, FAS, ENPP7) in both mouse and human islets at normal physiological values did not indicate acute GSIS changes. At higher levels (>1 nM), IL-18Ra and RTN4R increased apoptosis in mouse islets (IL-18Ra ~3-fold; NogoR > 15-fold). In vivo, in experimental models on a high-fat high-sucrose (HFHS) diet, RTN4R improved glucose tolerance without affecting acute insulin secretion, while in the db/db model it worsened fasting glucose levels and reduced insulin sensitivity.

#### 3.3.2. Human Clinical Associations—Circulating Nogob and Vascular Complications

Irakoze et al. [59] performed a retrospective single-center study in which fasting serum levels of NogoB and their association with vascular complications in individuals with diabetes were analyzed. The study included 733 subjects divided into three groups: healthy controls (NC, n = 300), patients with diabetes without vascular complications (DM, n = 68), and patients with diabetes with macrovascular and/or microvascular complications (DM + VC, n = 365). Serum levels of NogoB were measured using ELISA. Statistical analysis included multivariate logistic regression models that were gradually adjusted for demographic and clinical covariates, and ROC analyses for assessment of discriminative ability. Immunohistochemical analysis of subcutaneous tissue biopsies obtained from diabetic foot ulcer areas compared with control samples provided additional data, while in vitro experiments on HUVEC cells supported possible mechanisms.

Average serum NogoB concentrations were highest in controls, lower in the DM group, and lowest in the DM + VC group, with statistically significant differences (*p* < 0.001). In multivariate models, lower circulating NogoB remained strongly associated with the presence of vascular complications even after adjustment for clinical covariates (e.g., OR ≈ 0.63, *p* < 0.001; adjusted models OR ≈ 0.65, *p* < 0.001 when comparing DM + VC vs. NC). ROC analysis showed a high ability to distinguish DM + VC from NC (AUC = 0.927, 95% CI 0.909–0.945; selected threshold 28.82 ng/mL, sensitivity 83.6% and specificity 83.7%) and moderately high ability to distinguish DM + VC from DM (AUC = 0.840, threshold 27.68 ng/mL). Immunohistochemical staining of subcutaneous skin biopsy samples from the DFU area showed reduced expression of NogoB compared with control samples (*p* < 0.01). In vitro data in HUVEC cells indicated that high-glucose and cholesterol conditions reduced NogoB expression and were associated with indicators of endothelial dysfunction, which the authors proposed as a possible mechanistic framework.

## 4. Discussion

### 4.1. Interpretation of the Main Findings from the Results

Taken together, the data suggest that RTN4 isoforms (NogoA/B/C) and their receptors act through multiple, interrelated pathophysiological mechanisms that together contribute to glucoregulatory dysfunction and the development of vascular complications in T2D.

#### 4.1.1. Pancreas—Direct Effects on β-Cells—NogoA → Reduced Insulin Secretion

In pancreatic β-cells, NogoA is localized in the ER, as well as in vesicular membranes, and can therefore cause disturbances in β-cell function [43]. It can disrupt their function through two related mechanisms, which ultimately lead to decreased mobilization and fusion of insulin granules. One mechanism by which NogoA influences pancreatic β-cells involves intracellular modulation of ER-Golgi organization and secretory granule biosynthesis. The second mechanism refers to extracellular activation of complexes mediated by NogoR signaling, which are responsible for remodeling of the actin cytoskeleton, as well as for fusion through the RhoA/ROCK cascade. The intracellular mechanism implies that NogoA, as a protein involved in reticulon shaping, binds to domains located on the ER, which can significantly influence ER membrane morphology. In addition, it plays an important role in the separation of vesicles directed toward the Golgi apparatus. Through changes in ER morphology in β-cells, the passage of proinsulin through the secretory pathway can be slowed or hindered, thereby prolonging its retention within the ER, which in turn promotes a mild, chronic stress response. This has the following consequences: (1) an increased proinsulin-to-insulin ratio inside the cell and in secretion; and (2) decreased activity of prohormone convertases (for example, PCSK1/PC1/3), together with changes in the luminal environment, oxidative stress, and disruption of disulfide bond formation. Such inadequate and impaired processing leads to a decrease in the number of properly filled and mature secretory granules (reduced density and heterogeneous granule size, as seen by electron microscopic analysis of tissue samples). This reduction directly decreases the number of granules available for the rapid initial response and those in reserve required during subsequent glucose-stimulated insulin secretion [49,51].

On the other hand, interactions occurring within the extracellular space of islets or through vesicular secretion can lead to activation of the extracellular domain of NogoA. In this way, RTN4R is activated, together with related coreceptors found in β-cells themselves. The resulting NogoA-receptor complex triggers RhoA to switch to its active GTP-bound state and subsequently activates ROCK kinase. Literature data then indicate that excessive activation of RhoA/ROCK creates a physical barrier that limits granule transport. As can be seen, the literature data suggest that the combination of an intracellular lack of mature and properly filled granules with cytoskeletal blockage responsible for their mobilization may cause a gradual decline in secretory capacity. In practical terms, this could mean that a reduction in the first-phase response and weakening or incompleteness of the second-phase response occur. Bonal et al. [43] suggest that such changes can manifest clinically and experimentally as impaired glucose-stimulated insulin secretion and worsening glucose tolerance (see Table 2). Therefore, according to the available literature data and the findings of original research, it can be concluded that reduced GLP-1 levels weaken intestinal support for β-cells, further decreasing the rapid initial response. As a result, glycemic control significantly worsens, contributing to diabetes progression or the development of complications.

#### 4.1.2. Digestive Tract (Gut)—NogoB → proGCG/PCSK1 → Reduced GLP-1

NogoB is recognized as an integral ER membrane protein found in intestinal enteroendocrine L-cells, and it is believed to modulate the intestinal incretin axis through binding to proGCG, thereby affecting the production of active GLP-1, and/or through the formation of heterocomplexes with ER luminal chaperones. Literature data further indicate that these changes may lead to alterations in intracellular transport of proGCG from the ER [36,49]. Review of the literature suggests that these interactions have several synergistic implications that may lead to reduced conversion of proGCG to active GLP-1. First, research indicates that binding of proGCG to NogoB and/or to chaperones can cause conformational changes and make it more difficult for PCSK1 (PC1/3) to access specific processing sites on proGCG. Such conditions lead to reduced efficiency of proteolytic cleavage required for the production of active GLP-1 peptides. Another possible common mechanism is that, by binding to the ER membrane, RTN4B can slow the transfer of proGCG from the ER to the Golgi apparatus. In this way, the probability that molecules will be directed toward ER-associated degradation (ERAD) or alternative degradation pathways increases. It has been suggested that this further reduces the amount of processed peptide available for secretion [50]. In addition, available literature data have shown that changes in the ER microenvironment, especially calcium balance and oxidative status, can significantly affect disulfide bond formation. Therefore, proteolytic cleavage of proGCG by PCSK1 is slowed or reduced due to changes occurring in the interactions between RTN4B and ER chaperones [49]. Taken together, all described mechanisms indicate that less proGCG is properly converted into active GLP-1. This further leads to significant incretin deficiency and weaker glucose-dependent activation of β-cells, thereby contributing to postprandial hyperglycemia (shown in Table 2).

**Table 2:** Overview of key original studies; their methods and findings.

Study (Year)	Model/Methods	Main Finding	Mechanism
Bonal CB et al., 2013 [43]	Mice with NogoA downregulation; isolated islets of Langerhans; in vitro GSIS; in vivo glycemic tests; RTN4 immunohistochemistry	Downregulation of NogoA → increased GSIS; better glycemic control; expression of NogoA in β cells	NogoA negatively modulates insulin secretion; target modulation can improve β cell function
Liu X et al., 2014 [57]	Diabetic rats; quantification of RTN4R expression; RhoA/ROCK activities; TUNEL analysis; RTN4R antagonists	Increased expression of RTN4R → increased activation of Rho/ROCK and apoptosis of ganglion cells; RTN4R blockade reduces apoptosis	RTN4R → RhoA/ROCK activation → proapoptotic signals
Yang Q. et al., 2021 [58]	Diabetic models (mouse/rat); local siRNA reduction in NogoB expression in the retina; Evans Blue/fluorescence assay BRB; Western blot for Src/PI3K/Akt/ERK; inflammatory markers	Reduced expression of NogoB → reduced BRB permeability; decreased activation of Src/ERK; minor inflammation of the retina	NogoB contributes to Src/PI3K/Akt/ERK-mediated BRB dysfunction; inhibition reduces complications
Chen Y. et al., 2021 [44]	Hepatocyte-specific RTN4BR KO/overexpr.; GTT/ITT; triglyceride/cholesterol measurement; Western blot for GRP78/CHOP; phosphorylation of Akt; pathohistological analysis of liver samples	Loss of RTN4BR → increased ER stress marker, decreased Akt phosphorylation, worsening insulin sensitivity and dyslipidemia	RTN4BR is important for ER homeostasis and insulin signaling in the liver
Gong K. et al., 2024 [35]	In vitro (enterocytes); Immunohistochemical analysis; co-IP NogoB/proGCG; siRNA/KO in cells and db/db mice; PCSK1 enzyme assays; GLP-1 ELISA; GTT	NogoB binds proGCG → reduced PCSK1 processing → lower GLP-1	NogoB in the ER directly inhibits proGCG processing and reduces GLP-1

Abbreviations: NogoA—Nogo A protein; GSIS—glucose-stimulated insulin secretion; RTN4R—Nogo receptor; RhoA/ROCK—Rho-associated protein kinase; TUNEL—Terminal deoxynucleotidyl transferase; dUTP—nick end labeling; BRB—blood–retinal barrier; GTT/ITT—Glucose Tolerance Test/Insulin Tolerance Test; GRP78/CHOP—a chaperone, pro-survival/a transcription factor, pro-apoptotic; RTN4BR—Nogo B receptor; ER—endoplasmic reticulum; PCSK1—Proprotein Convertase Subtilisin/Kexin type 1; GLP-1—Glucagon-like peptide-1; proGCG—proglucagon.

#### 4.1.3. Regulation of Insulin Signaling—NogoB Receptors in the Liver

The study conducted by Chen et al. [44] on experimental models in which they induced a decrease in RTN4BR expression in the liver supports findings from previous studies—deficiency of the RTN4BR in hepatocytes increases ER stress, leads to a decrease in Akt phosphorylation, and worsens insulin resistance. Conversely, overexpression of RTN4BR is associated with a significant improvement in insulin signaling and a reduction in ER stress markers [44] (see Table 2). Thus, there is a strong connection between RTN4BR, dolichol-dependent glycosylation, membrane lipid homeostasis, activation of ER stress, and the unfolded protein response (UPR). This further suggests that any deviation from this relationship could lead to the development of inflammatory processes, lipid accumulation, as well as changes in serine phosphorylation of IRS1/2 (insulin receptor substrates 1 and 2), which further causes inhibition of the PI3K/Akt signaling pathway and thus contributes to the development of insulin resistance [25,44,55,56].

#### 4.1.4. Retina—RTN4R and ROCK; RGC Apoptosis

Liu et al. [57], based on the well-known fact that Nogo receptors are potent inhibitors of axonal growth and promoters of neurodegenerative processes, hypothesized in their research that they may also be involved in retinal neurodegeneration in experimental models of diabetes, i.e., rats, through similar mechanisms. In their study, they analyzed in detail the expression of RTN4R using immunohistochemistry and Western blot. In addition, apoptosis of retinal ganglion cells was evaluated using the TUNEL assay, and the activity of the RhoA/ROCK signaling pathway was measured. In order to demonstrate causality between apoptosis of nuclei in retinal layers and RTN4R, pharmacological or molecular antagonists of RTN4R were used in part of the experiment to inhibit RTN4R function. The results of this research clearly indicated a significant increase in RTN4R expression in the retina of animals with DM, where it was most strongly expressed in the retinal ganglion layer. This phenomenon was accompanied by an increased activation of the RhoA/ROCK signaling pathway in the retina of experimental rats with DM, as well as an increased number of TUNEL-positive ganglion cells in the retina. These findings indicate increased apoptosis of retinal ganglion cells, which are key neurons responsible for transmission of visual signals, in the presence of diabetes. Exceptional clinical importance, in terms of identifying the RTN4R–RhoA/ROCK axis as a potential therapeutic target for preventing or slowing neurodegeneration in diabetic retinopathy, was also demonstrated by the part of the study in which interventions aimed at blocking RTN4R function were applied. Namely, the applied methods for blocking RTN4R function led to reduced activation of both the RhoA/ROCK signaling pathway and apoptosis of retinal ganglion cells in experimental animals with DM. These findings unequivocally suggest a functional connection between increased RTN4R expression and neurodegenerative processes occurring in the diabetic retina [57].

#### 4.1.5. Vascular Effect: NogoB/RTN4BR → Src → PI3K/Akt/ERK and RhoA → ROCK

The formation of the RTN4BR complex initiates a complex signaling cascade that leads to disruption of endothelial barrier integrity, thereby significantly facilitating neurovascular dysfunction. The binding of NogoB and Nogo receptors recruits Src kinase and further activates two signaling pathways, namely PI3K → Akt and MAPK/ERK. In addition, it can cause phosphorylation of very important components—adhesion molecules. Namely, it is suggested that phosphorylation leads to internal degradation and breakdown of adhesion molecules within the retina and vascular endothelium. In addition to phosphorylation of adhesion molecules, phosphorylation of other target proteins such as occludin, claudin, and ZO-1, which are essential for tight junctions, also occurs. Conducted studies have shown that activation of RhoA/ROCK leads to changes in the actin cytoskeleton (actin reorganization), thereby increasing endothelial cell contractility, which is one of the factors responsible for increased permeability of blood vessel walls. These results indicate that inhibitors of RhoA/ROCK or the RTN4R/Src signaling pathway can affect blood vessel wall permeability, i.e., reduce permeability and thereby decrease edema, inflammation, and neuronal degeneration occurring as part of diabetic complications (retinopathy, neuropathy) (see Table 2) [57,58].

In human endothelial cell models, specifically in the HUVEC study, it was shown that extremely high glycemia and/or cholesterol levels lead to a decrease in endogenous NogoB. This subsequently induces inflammation, i.e., production of cytokines and TGF-β, which in turn promotes EndMT and leads to loss of expression of endothelial markers such as CD31 and eNOS, as well as increased expression of mesenchymal markers such as α-SMA and collagen I [59]. Results from all studies clearly indicate that RTN4 knockdown worsens inflammation and EndMT, while also causing a decrease in angiogenic function and cell viability. The exact opposite effect is observed with RTN4 overexpression. Research has shown that RTN4 overexpression leads to reduction in the TGF-β/p-Smad2/3 signaling pathway, reduction in EndMT, and improvement of endothelial function, i.e., improved cell viability and regeneration [59,60].

#### 4.1.6. Renal Protection

In diabetic kidney models, elevating circulating sNogoB reduced albuminuria, mitigated glomerular hyperfiltration, and preserved podocyte number and glycocalyx integrity. These effects were associated with dampening of VEGF-A/VEGFR2 signaling and normalization of eNOS/AKT/GSK3β phosphorylation, and sNogoB was shown to interact with RTN4BR on glomerular endothelial cells, supporting a paracrine regulatory mechanism for glomerular permeability [60]. These findings indicate that soluble Nogo variants can modulate glomerular vascular signaling and protect against diabetic glomerular injury.

#### 4.1.7. Molecular Structure and Genetic Loss-of-Function

Park et al. [55] and Edani et al. [56] indicate that, acting as an important component of cis-prenyltransferase (cis-PTase) and as a regulator of dolichol biosynthesis, RTN4BR plays an essential role in maintaining ER membrane and lipid balance. In their research, this group of researchers further states that RTN4BR stabilizes dehydrodolichyl diphosphate synthase and affects the catalytic activity of cis-prenyltransferase, thereby stimulating dolichol production and proper protein glycosylation. However, disturbances in RTN4BR function lead to decreased efficiency of dolichol-dependent glycosylation. In addition, changes occur in the phospholipid and sterol composition of the ER membrane. Park et al. and Edani et al. further suggest that all these changes and phenomena significantly compromise membrane integrity and lipid homeostasis [55,56].

According to Samuel and Shulman [24], the lipid imbalance occurring in the ER triggers metabolic adaptation in the form of increased de novo lipogenesis (for example, through activation of SREBP1c). In addition, they further state that this process occurs simultaneously with reduced β-oxidation and decreased VLDL secretion. The aforementioned researchers believe that these changes cause accumulation of triglycerides within hepatocytes, which in turn increases production of lipotoxic lipid mediators, whereby this lipid imbalance further increases ER stress and reduces the ability to process proteins [24]. Activation of the UPR is caused precisely by disturbed ER homeostasis, as well as accumulation of inadequately glycosylated proteins, whereby persistently activated UPR shifts from the adaptive phase to a proinflammatory and proapoptotic phase, during which other signaling pathways are activated (for example, NF-κB), thereby linking ER stress with inflammatory responses in the liver [25]. All these findings support the fact that there is a decrease in Akt phosphorylation through increased phosphorylation of IRS1/2, the proteins responsible for transmitting signals from the insulin/IGF-1 receptor to the interior of the cell, as well as phosphorylation of serine residues on these proteins [24,25].

**Figure 2:**
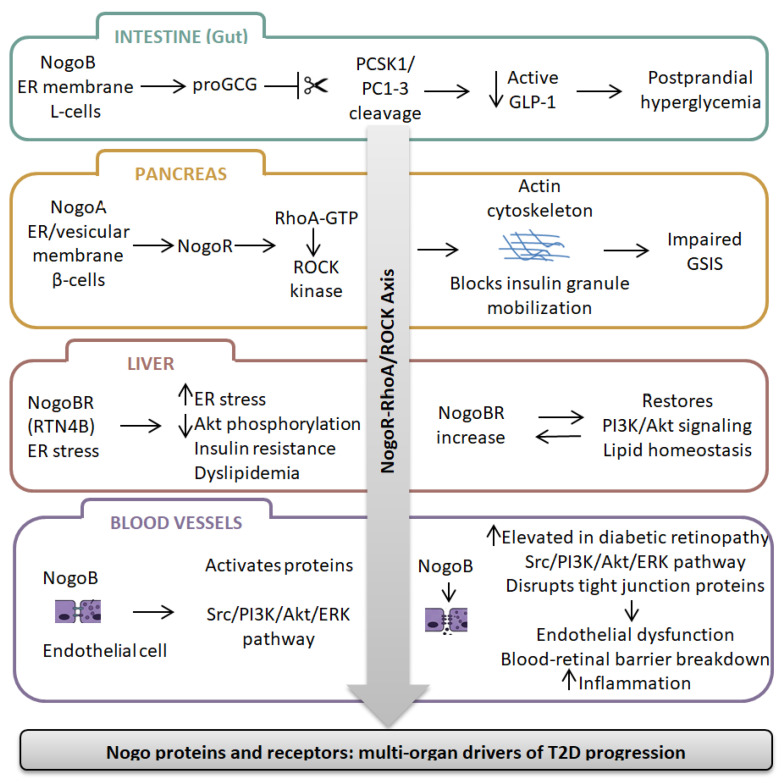
Multi-organ role of Nogo proteins and receptors in diabetes mellitus.

The roles of Nogo proteins and receptors in DM, i.e., the role of the RTN4R–RhoA/ROCK pathway, including mechanisms involving the pancreas, liver, intestine, and vasculature, are shown in Figure 2.

### 4.2. Human Studies

#### 4.2.1. Cohort Multi-Omics Discovery and Validation

Modern proteomic analyses from the RHAPSODY study identified RTN4 as one of the proteins associated with faster progression of T2D, representing a significant marker of worsening glycemic control, i.e., an earlier transition to insulin therapy. Findings from this study showed that, even in human islets of Langerhans exposed to high glucose concentrations, RTN4R promoted apoptosis. The results of this work suggest that proteomic signals require further quantitative validation, and that Mendelian randomization analysis for RTN4R did not demonstrate causality, although GDF15 and several other proteins were supported by MR findings. These findings, obtained from independent cohorts, support the concept that Nogo proteins and Nogo receptors may be useful in assessing disease severity and outcomes, i.e., that they may possess prognostic value [33].

#### 4.2.2. Human Clinical Associations—Circulating NogoB and Vascular Complications

In a large cohort study conducted by Irakoze et al., it was demonstrated that serum NogoB levels differed significantly between individuals with T2D and vascular complications and individuals with T2D without vascular complications [59]. Such findings remained significant after multivariate analyses, including adjustment for age, sex, risk factors, and medications used by the participants. In addition, ROC analysis performed in the study demonstrated significant discriminatory ability for differentiating patients with T2D and vascular complications from the control group, as well as for differentiating T2D patients with vascular complications from T2D patients without vascular complications. Furthermore, in a subgroup of patients with T2D and diabetic foot ulcers, the authors observed an additional decrease in serum NogoB levels, as well as reduced NogoB expression in subcutaneous tissue demonstrated by immunohistochemistry, suggesting an important connection between local and systemic changes. Findings from earlier studies reporting opposite effects, such as increased Nogo levels in proliferative retinopathy, should also be acknowledged, suggesting possible heterogeneity depending on disease type and measurement methodology [59].

A detailed overview of the experimental evidence for the associated signaling pathways and outcomes is provided in Table 3.

### 4.3. Preclinical Mechanisms vs. Evidence in Humans

Preclinical evidence in mice and cell models consistently shows that manipulation of RTN4/Nogo isoforms and their receptors significantly affects GLP-1 secretion, GSIS, ER stress, and vascular permeability. Genetic and pharmacological approaches (knockout/knockdown, overexpression, siRNA, neutralizing antibodies, RhoA/ROCK inhibitors, and RTN4R blockers) reproducibly improve or normalize these functions in diabetes models, providing a robust mechanistic framework and potential therapeutic targets [24,25,35,43,44,49,50,55,56,57,58]. Mechanistic data are tissue-specific: NogoB disrupts proGCG/PCSK1 processing and reduces active GLP-1 (enterocytes) [35,49,50]; NogoA, together with ER/Golgi dysfunction and RTN4R-mediated activation of RhoA/ROCK, disrupts the cytoskeleton and reduces GSIS (β-cells) [43,49,50,51,52,53,54]; RTN4BR affects dolichol-dependent glycosylation, increases ER stress, and decreases p-Akt, leading to insulin resistance (liver) [24,25,44,55,56]; and NogoB/RTN4BR activate Src → PI3K/Akt/ERK and RhoA → ROCK signaling, which disrupts tight junctions, increases vascular permeability, and contributes to retinopathy (vascular/neuronal models) [49,57,58,60]. Intervention experiments targeting these pathways usually improve functional outcomes (higher GLP-1 levels, improved GSIS, reduced ER stress, and decreased vascular permeability).

Human evidence is complementary but heterogeneous and context-dependent. Proteomics and cohort analyses identified RTN4/Nogo components (e.g., RTN4R and circulating NogoB) as biomarkers associated with faster T2D progression and the presence of vascular complications, while quantitative serological differences and ROC analyses indicated potential prognostic value [33,59]. However, the direction and localization of the findings vary (e.g., elevated RTN4 in some cases of proliferative retinopathy versus decreased NogoB in serum or ulcer tissue), likely reflecting differences in disease stage, sampling (serum vs. tissue), measurement methodology, and organ-specific compensatory responses [59,60]. A key limitation lies in MR analysis for RTN4R. MR analysis for RTN4R did not confirm causality, which weakens the argument for a direct causal role of RTN4R in humans and indicates that proteomic associations alone are insufficient to infer therapeutic value without additional genetic and functional validation [33].

Thus, coherent and translatable mechanisms identified in preclinical models justify further investigation of the RTN4 axis as a therapeutic target, but human data still require rigorous quantitative validation, functional correlation in relevant tissues, and studies designed to assess causality before clinical application. Although preclinical work shows that manipulation of the Nogo axis can have functional consequences relevant to metabolism and vascular homeostasis, translation of these findings to patients requires standardized, quantitatively validated protein measurements (isoform-specific ELISA), longitudinal human data monitoring protein changes before and after development of complications, genetic integration through cis-pQTL, colocalization, and MR analyses to exclude reverse causality and determine concordance between genetics and proteomics, and tissue-specific confirmation in human biopsies (e.g., retina, pancreatic islets, and liver). Future work should also aim to reproduce and quantitatively validate proteomic findings in independent human cohorts; standardize measurement methods (clear differentiation of isoforms, serum vs. tissue, validated antibodies/assays); conduct additional MR analyses using larger and more robust genetic instruments and/or multivariate approaches to verify causality; examine potential side effects of systemic modulation of the RTN4 system (tissue specificity, compensatory mechanisms); and perform functional ex vivo experiments on human tissues (islets, hepatocytes, endothelia) in order to verify mechanisms established in experimental models.

### 4.4. Heterogeneity and Contradictions—Possible Sources

Apparent contradictions across studies and sample types are substantial and must be addressed explicitly. Likely contributing factors include the following:

*Tissue and isoform specificity*: RTN4 encodes multiple isoforms (NogoA, NogoB) with differing subcellular localizations (ER membrane vs. extracellular/secreted fragments) and receptor/co-receptor interactions; the same family may exert opposing effects depending on isoform and tissue (pancreas vs. gut vs. liver vs. endothelium) [35,43,44].

*Model and disease-stage dependence*: Experimental outcomes vary by model and disease stage (HFHS, HGD, STZ/HFD, db/db). For example, RTN4R improved OGTT in HFHS mice but worsened metabolic parameters in db/db mice; NogoB knockdown disrupted BRB integrity in healthy retina yet ameliorated BRB breakdown in diabetic retina [33,57,58]. Disease stage (early vs. advanced) likely modifies the direction of the observed effects.

*Assay, antibody, and platform variability*: SomaScan proteomics, different ELISA kits, SPR/ELISA binding assays, tissue IHC, and mass spectrometry provide non-identical quantitative readouts. Antibody epitope specificity (isoform/fragment selectivity) further contributes to discordant measurements between studies and biological matrices (plasma vs. vitreous vs. tissue).

*Dose/concentration and exposure*: Functional effects are concentration-dependent. Proteomic prioritization experiments reported that supraphysiological RTN4R concentrations induced apoptosis in islets (>1 nM), whereas physiological levels had no acute GSIS effect [33]. Therapeutic modulation may therefore follow non-linear dose–response relationships.

*Confounding in human cohorts*: Comorbidities, medications, metabolic status, renal clearance, timing of sample collection, and selection bias may confound associations between circulating Nogo proteins and clinical outcomes [59]. Cross-sectional analyses cannot establish temporality.

*Genetic and post-translational modulation*: Genetic variants (e.g., RTN4BR/NUS1 biology) and post-translational modifications (glycosylation, cleavage into secreted fragments) alter protein function and measurement, while lack of isoform-specific genetic proxies limits MR analyses [55,56]. Within large multi-omics studies that included RTN4/RTN4R signaling (e.g., RHAPSODY/Slieker et al.), MR analyses have thus far failed to support a direct causal effect of RTN4R on glycemic deterioration in available data sets [33]. This negative MR finding reduces confidence in a direct causal interpretation of proteomic associations and preclinical modulation studies. Specifically, alternative explanations remain possible: (i) RTN4/RTN4R proteomic signals may represent secondary disease-related changes (reverse causation); (ii) signals may reflect compensatory or context-dependent responses; or (iii) the instruments used for MR analyses may have lacked sufficient statistical power or failed to adequately reflect tissue-specific RTN4/RTN4R expression. Therefore, a negative MR result does not exclude biological relevance in all contexts, but it justifiably reduces confidence in the direct causality of proteomic observations [33].

To increase the statistical power and validity of MR analyses for RTN4/RTN4R, future studies should (a) expand MR analyses using larger and more robust genetic instruments, with emphasis on strong cis-pQTLs targeting RTN4/RTN4R levels in serum or tissues, thereby reducing the possibility of horizontal pleiotropy and improving reliability of MR estimates [33]; (b) perform colocalization analyses between cis-pQTL/eQTL and GWAS signals for glycemic phenotypes to determine whether the same genetic signal simultaneously influences protein levels and clinical outcomes; and (c) direct functional experiments toward genetic findings by using genetically informed indicators (e.g., variants associated with pQTLs) to select isoforms and tissues for targeted perturbation in human-relevant models (human islets, organoids, tissue-targeted knockdown/overexpression) [33].

### 4.5. Implications for Biomarker Development

Proteomics and cohort-derived signals (RTN4R, circulating NogoB) justify targeted quantitative validation. Required steps include development of isoform-specific quantitative assays (validated ELISA or mass spectrometry methods) with defined LLOQ/ULOQ and reference standards; longitudinal cohort studies with serial sampling to assess temporal predictive value (time-to-insulin initiation, incident vascular events); derivation and external validation of ROC thresholds; integration of cis-pQTL and MR analyses to assess causality; and functional confirmation in human cellular and tissue models. Because differences in measurement platforms and antibody epitopes contribute substantially to heterogeneity, assay harmonization and standardization are essential prerequisites.

### 4.6. Qualitative Assessment of Evidence and Limitations

Available human studies provide strong support for an association between Nogo proteins (primarily NogoB) and Nogo receptors and the occurrence of vascular complications and adverse vascular outcomes in individuals with T2D. However, these findings are predominantly retrospective in nature, and large prospective studies are necessary for confirmation. Proteomic analyses from RHAPSODY additionally suggest that RTN4R may represent a marker of diabetes progression, although these findings also require rigorous quantitative validation. Further studies should include ELISA-based quantification and comprehensive verification of causality through genetic analyses. Experimental preclinical studies have consistently demonstrated mechanistic effects, but translation from experimental models to human application requires caution because the observed effects appear to depend on protein isoform and exposure levels. Furthermore, heterogeneity among studies—for example, increased versus decreased retinal expression in different contexts—highlights the need for clearly defined patient populations and standardized measurement methodologies before Nogo proteins can be considered reliable prognostic biomarkers. Taken together, Nogo proteins and Nogo receptors represent promising candidates for clinical biomarkers of vascular complications in diabetes, but strict quantitative validation and confirmation of predictive and clinical value through prospective studies remain necessary before clinical implementation.

### 4.7. Recommendations for Future Research

Measurement standardization is essential; specifically, quantitative validation studies are required to harmonize standards for assessment of Nogo proteins and Nogo receptors (e.g., ELISA) across laboratories, followed by rigorous reproducibility analyses. In addition, findings should be replicated in prospectively followed individuals with T2D, with repeated measurements performed to evaluate predictive value for the development of complications and for the period preceding insulin initiation. Furthermore, future studies should include additional in vivo investigations to define therapeutic thresholds and evaluate risks associated with modulation of the Nogo axis, including determination of the effects of NogoR modulation on pancreatic islets.

## 5. Conclusions

Preclinical evidence supporting a role of Nogo/RTN4 in β-cell function, incretin processing, ER homeostasis, and vascular pathology is strong and coherent, but by itself remains insufficient for clinical application without confirmation in human genetic and longitudinal studies. Proteomic and serological associations from human studies provide important translational insights, but previously weak or negative Mendelian randomization results in existing cohorts require caution when interpreting causality; future research should integrate robust genetic instruments (preferably cis-pQTLs), colocalization analyses, and functional validation. If consensus between genetic and functional evidence is achieved—i.e., robust cis-pQTLs with signal colocalization and reproducible human-relevant functional effects—RTN4/RTN4R/RTN4BR may become promising candidates for further development as prognostic biomarkers and/or therapeutic targets in T2D.

## Figures and Tables

**Table 1:** Consolidated table organized by evidence category (all studies, sample sizes, methods, and outcomes).

Author (Year)	Type/Model	Sample Size (n)	Tissue/Sample	Method	Key Outcome	Inclusion Reason
**Preclinical study**						
Bonal CB (2013) [43]	Mouse/WT vs. NogoA KO; db/db + antibody	n = 5–10	Islets, pancreas, plasma	WB, IHC, ELISA, GSIS, GTT	Glycemia KO 8.48 vs. WT 10.66 mmol/L (*p* = 0.04)	Mechanistic: Nogo-A → β-cell/GSIS
Liu X (2014) [57]	Rat/STZ diabetic rat retina	40 rats	Retina (RGCs)	siRNA, IHC, WB, TUNEL	ROCK + RGCs: 35.2% (DM) vs. 11.6% (siNgR) *p* < 0.01	Mechanistic: RTN4R → RhoA/ROCK → RGC apoptosis
Gong K (2024) [35]	Mouse-cell/enterocytes; db/db; HFD/STZ	n = 6/group	Intestine, serum GLP-1, pancreas	siRNA KD, GTT, ITT, clamp, IP-MS	KD → ↓ BW, ↓ glycemia, ↑ GLP-1, improved GTT	Mechanistic: NogoB → proGCG/PCSK1 → GLP-1
Chen Y (2021) [44]	Mouse/RTN4BRhepKO; AAV9 NGBR liver	Varies	Liver, skeletal muscle, plasma	AAV OE, WB, qPCR, histology, clamp	KO: fasting glucose ↑~40%; AAV9: ↓ NEFA/TG	Mechanistic: RTN4BR → ER stress/AKT → insulin sensitivity
Yang Q (2021) [58]	Rat-retina/STZ diabetic rat; RGCs; HUVEC	100 rats; 14 DM pts (ELISA)	Retina, plasma/vitreous, HUVEC	ELISA, AAV siRNA, WB, qRT-PCR, permeability	NogoB silencing → ↓ BRB impairment via Src/PI3K/Akt/ERK	Mechanistic: NogoB → BRB/retinal vascular
Hernandez-Diaz I (2019) [60]	Mouse/STZ diabetic; renal cortex; glomeruli	Not listed	Plasma, renal cortex, glomeruli, kidney	AAV sNogo-B OE, ELISA, EM, histology, urine albumin	sNogoB ↑~12-fold; albuminuria ↓~50%	Mechanistic: sNogo-B → renal vascular protection
**Molecular/structural study**						
Park EJ (2014) [55]	Genetic-mouse/NgBR KO mice; MEFs; human exome	Genetic lines; in vitro replicates	Mouse organs, fibroblasts, cell lines	Conditional KO, filipin staining, cis-PTase, exome seq	Homozygous KO lethal < E6.5; iKO MEFs: ↑ free cholesterol, ↓ esterification	Mechanistic: NgBR → dolichol biosynthesis → N-glycosylation
Edani BH (2020) [56]	Structural/RTN4BR/DHDDS complex (recombinant)	N/A (crystallography)	Recombinant DHDDS + RTN4BR fragments; crystals	X-ray crystallography (2.3 Å), cis-PTase assays, mutagenesis, TLC/HPLC	Structure at 2.3 Å; PI stimulates activity; key mutations characterized	Structural: RTN4BR/DHDDS → dolichol → ER N-glycosylation
**Human: Chort multi-omics**						
Slieker RC (2023) [33]	Proteomic cohort/Multi-omics human cohorts	Discovery n = 2973; Validation n = 2668	Human plasma (metabolomics, lipidomics, proteomics)	UHPLC-MS/MS, Lipotype MS, proteomics	AADA HR = 1.11 (pFDR = 0.03); homocitrulline HR = 1.12 (pFDR = 0.03); RTN4/RTN4R → glycemic deterioration	Human biomarker: RTN4/RTN4R → T2D progression
Slieker (2021, preprint) [34]	Proteomic cohort/IMI-RHAPSODY: DCS, GoDARTS, Atherosclerosis	DCS n = 1267; GoDARTS n = 900	Human plasma (metabolomics, lipidomics, SomaScan proteomics)	UHPLC-MS/MS, Lipotype MS, SomaScan	RTN4/RTN4R candidate biomarkers identified for glycemic progression	Supporting proteomics: RTN4/RTN4R biomarker discovery (preprint)
**Human: Clinical associations**						
Irakoze L (2026) [59]	Human cohort-serology/T2D ± vascular complications; NC	DM + VC n = 365; DM n = 68; NC n = 300; total n = 733	Fasting venous serum; HUVEC supernatants	Serum NogoB ELISA (Jiubang QZ-17338), IHC, HUVEC assays	NogoB ↓ in DM + VC; ROC AUC = 0.927 (95% CI 0.909–0.945)	Translational: lower NogoB → vascular complications in T2DM

Abbreviations: AAV—adeno-associated virus; AUC—area under the curve; BRB—blood–retinal barrier; CI—confidence interval; db/db—leptin receptor-deficient diabetic mouse model; DCS—Diabetes Care System cohort; DM—diabetes mellitus; DM + VC—diabetes mellitus with vascular complications; ELISA—enzyme-linked immunosorbent assay; EM—electron microscopy; ER—endoplasmic reticulum; GEC—glomerular endothelial cell; GLP-1—Glucagon-like peptide-1; GoDARTS—Genetics of Diabetes Audit and Research in Tayside Scotland; GSIS—glucose-stimulated insulin secretion; GTT/ITT—Glucose Tolerance Test/Insulin Tolerance Test; HFD—high-fat diet; HUVEC—human umbilical vein endothelial cell; IHC—immunohistochemistry; IP-MS—immunoprecipitation–mass spectrometry; KD—knockdown; KO—knockout; LC-MS/MS—liquid chromatography–tandem mass spectrometry; MAG—myelin-associated glycoprotein; MEFs—mouse embryonic fibroblasts; NC—normal controls; NEFA—non-esterified fatty acids; RTN4BR—Nogo-B receptor; RTN4R—Nogo receptor; NogoA—Nogo A protein; NogoB—Nogo B protein; OE—overexpression; PCSK1—Proprotein Convertase Subtilisin/Kexin type 1; PI3K—phosphoinositide 3-kinase; qPCR—quantitative polymerase chain reaction; qRT-PCR—quantitative reverse transcription polymerase chain reaction; RGC—retinal ganglion cell; RhoA/ROCK—Rho-associated protein kinase; ROCK—Rho-associated protein kinase; siNgR—small interfering RNA targeting Nogo receptor; siRNA—small interfering RNA; SomaScan—aptamer-based proteomic platform; SPR—surface plasmon resonance; Src—proto-oncogene tyrosine-protein kinase Src; STZ—streptozotocin; T2D—type 2 diabetes; TLC/HPLC—thin-layer chromatography/high-performance liquid chromatography; TNF-α—tumor necrosis factor alpha; TUNEL—Terminal deoxynucleotidyl transferase dUTP nick end labeling; UHPLC-MS/MS—ultra-high-performance liquid chromatography–tandem mass spectrometry; VEGF—vascular endothelial growth factor; WB—Western blot; WT—wild type.

**Table 3:** Signaling pathways, experimental evidence, outcomes, and implications.

Signaling Pathway/ Molecules	Example of Experiments	Organisms/ Models	Outcomes	Possible Therapeutics Implications
NogoB—proGCG—PCSK1—GLP-1	IP and KO/siRNA; increase in GLP 1 after NogoB downregulation; improved glycemia in db/db mice (PCSK1 enzyme assay; siRNA NogoB; GLP 1 ELISA; GTT)	In vitro enterocytes; db/db and WT mice (Gong et al.) [35]	Reduced proGCG processing; lower active GLP 1; improvement after NogoB downregulation	Local inhibition of NogoB in the intestine to increase endogenous GLP 1
RTN4BR (NUS1)—ER stress—Act	RTN4BR decrease → increased ER stress markers, decreased Akt phosphorylation; metabolic disorders (BiP/CHOP measurement; phospho Akt; lipid analysis)	Hepatocytes; mice with RTN4BR modification (Chen et al.) [44]	Increased UPR; decreased phospho Akt; insulin resistance	Enhancement of RTN4BR or UPR modulations in the liver
NogoA—β cells—GSIS	Downregulation of Nogo A → increased GSIS; Nogo A expression in islets	Isolated islets; KO/downreg mice (Bonal et al.) [43]	Increased GSIS after downregulation	Selective islet inhibition of NogoA
NogoB/RTN4R—Src/PI3K/Akt/ERK—BRB	Silencing NogoB reduces BRB permeability; regulation of Src/PI3K/Akt/ERK (RhoA/ROCK activity assays; TUNEL; RTN4R antagonists)	Diabetic models: mice/rats (Yang et al.) [58]	Rho/ROCK activation associated with apoptosis; reduction in apoptosis after blockade	RTN4R antagonists/RhoA/ROCK inhibitors for neuroprotection
RTN4R—RhoA/ROCK—apoptosis	Increased RTN4R associated with Rho/ROCK activation and TUNEL positive cells; RTN4R blockade reduces apoptosis (Src/ERK phosphorylation WB; Evans Blue; siRNA NogoB)	Diabetic rats (Liu et al.) [57]	Activation of kinases → dislocation of tight junctions → increased permeability	Src/ERK modulators; targeted silencing of Nogo B to preserve barriers

Abbreviations: Akt—protein kinase B; BiP/CHOP—binding immunoglobulin protein/C/EBP homologous protein; BRB—blood–retinal barrier; db/db mice—leptin receptor-deficient diabetic mice; ELISA—enzyme-linked immunosorbent assay; ER—endoplasmic reticulum; ERK—extracellular signal-regulated kinase; GLP-1—Glucagon-like peptide-1; GSIS—glucose-stimulated insulin secretion; GTT—Glucose Tolerance Test; IP—immunoprecipitation; KO—knockout; RTN4BR—NogoB receptor; RTN4R—Nogo receptor; NogoA—Nogo A protein; NogoB—Nogo B protein; PCSK1—Proprotein Convertase Subtilisin/Kexin type 1; PI3K—phosphoinositide 3-kinase; proGCG—proglucagon; RhoA/ROCK—Rho-associated protein kinase; siRNA—small interfering RNA; Src—proto-oncogene tyrosine-protein kinase Src; TUNEL—Terminal deoxynucleotidyl transferase dUTP nick end labeling; UPR—unfolded protein response; WB—Western blot; WT—wild type.

## Data Availability

No new data were created or analyzed in this study. Data sharing is not applicable to this article.

## Supplementary Materials

The following supporting information can be downloaded at: https://www.mdpi.com/article/10.3390/ijms27115124/s1.

## Abbreviations

The following abbreviations are used in this manuscript:

AADA	aminoadipic acid
AAV	adeno-associated virus
AAV9	adeno-associated virus serotype 9
AKT	protein kinase B
ALT	alanine aminotransferase
AMPK—AMP	activated protein kinase
Angpt1/2	angiopoietin 1/2
AST	aspartate aminotransferase
ATF4	activating transcription factor 4
AUC	area under the curve
BiP/GRP78	binding immunoglobulin protein/glucose-regulated protein 78
BMI	body mass index
BRB	blood–retinal barrier
Brn3a	brain-specific homeobox/POU domain protein marker of retinal ganglion cells
BW	body weight
CD31	cluster of differentiation 31
CDG	congenital disorder of glycosylation
CHOP	C/EBP homologous protein
CI	confidence interval
CNS	central nervous system
co-IP	co-immunoprecipitation
CRELD1	cysteine-rich with EGF-like domains 1
DCS	Diabetes Care System cohort
DFU	diabetic foot ulcer
DHDDS	dehydrodolichyl diphosphate synthase
DM	diabetes mellitus
eIF2α	eukaryotic initiation factor 2 alpha
ELISA	enzyme-linked immunosorbent assay
EM	electron microscopy
EndMT	endothelial-to-mesenchymal transition
ENPP7	ectonucleotide pyrophosphatase/phosphodiesterase 7
ER	endoplasmic reticulum
ERAD	ER-associated degradation
ERK	extracellular signal-regulated kinase
FAS	Fas cell surface death receptor
FITC	fluorescein isothiocyanate
FOXO1	forkhead box O1
GDF15	growth differentiation factor 15
GEC	glomerular endothelial cell
GFP	green fluorescent protein
GIP	glucose-dependent insulinotropic polypeptide
GLP-1	glucagon-like peptide-1
GPI	glycosylphosphatidylinositol
GSIS	glucose-stimulated insulin secretion
GSK3β	glycogen synthase kinase 3 beta
GTT	glucose tolerance test
GWAS	genome-wide association study
HbA1c	glycated hemoglobin
HDL	high-density lipoprotein
HEK293T	human embryonic kidney 293T cells
HepG2	human hepatocellular carcinoma cell line
HFD	high-fat diet
HFHS	high-fat/high-sucrose diet
HGD	high-glycemic diet
HR	hazard ratio
HUVEC	human umbilical vein endothelial cell
ICAM1	intercellular adhesion molecule 1
IDF	International Diabetes Federation
IHC	immunohistochemistry
IL-1β	interleukin 1 beta
IL-6	interleukin 6
IL-10	interleukin 10
IL-18Ra	interleukin 18 receptor alpha
IP	immunoprecipitation
IP-MS	immunoprecipitation–mass spectrometry
IPP	isopentenyl pyrophosphate/isopentenyl diphosphate
IRS1/2	insulin receptor substrates 1 and 2
ITT	insulin tolerance test
KD	knockdown
KO	knockout
LAMP1	lysosomal-associated membrane protein 1
LC-MS	liquid chromatography–mass spectrometry
LIMK	LIM kinase
LINGO1	leucine-rich repeat and Ig domain-containing Nogo receptor-interacting protein 1
MAG	myelin-associated glycoprotein
MAPK	mitogen-activated protein kinase
MEF	mouse embryonic fibroblast
MR	Mendelian randomization
MS	mass spectrometry
NC	normal control/healthy control
NEFA	non-esterified fatty acids
NF-κB	nuclear factor kappa B
NGBRhepKO	hepatocyte-specific NGBR knockout
RTN4R	Nogo receptor
NgR1/2/3	Nogo receptor 1/2/3
NogoA	Nogo A protein
NogoB	Nogo B protein
NogoC	Nogo C protein
RTN4BR	Nogo B receptor
NOS	Newcastle–Ottawa Scale
NUS1	NUS1 dehydrodolichyl diphosphate synthase subunit/NgBR gene
OE	overexpression
OR	odds ratio
p-AKT	phosphorylated AKT
p-AMPK	phosphorylated AMPK
p-eIF2α	phosphorylated eIF2α
p-GSK3β	phosphorylated GSK3β
p-INSR/IRS	phosphorylated insulin receptor/insulin receptor substrate
p75NTR	p75 neurotrophin receptor
PC1/3	prohormone convertase 1/3
PCSK1	proprotein convertase subtilisin/kexin type 1
PCR	polymerase chain reaction
PERK	protein kinase R-like ER kinase
pFDR	adjusted false discovery rate
PI	phosphatidylinositol
PI3K	phosphoinositide 3-kinase
PLA	proximity ligation assay
proGCG	proglucagon
pQTL	protein quantitative trait locus
PTase/cis-PTase	cis-prenyltransferase
QC	quality control
qPCR	quantitative polymerase chain reaction
qRT-PCR	quantitative reverse transcription polymerase chain reaction
RGC	retinal ganglion cell
RhoA/ROCK	Ras homolog family member A/Rho-associated protein kinase pathway
RO	receiver operating characteristic
ROCK	Rho-associated protein kinase
RT-PCR	reverse transcription polymerase chain reaction
RTN4	reticulon
RTN4B	reticulon 4B/NogoB
RTN4BR	reticulon 4B receptor/Nogo-B receptor
RTN4R	reticulon 4 receptor/Nogo receptor
RXG	arginine-X-glycine motif
siNgR	small interfering RNA targeting Nogo receptor
siRNA	small interfering RNA
SomaScan	aptamer-based proteomic platform
SPR	surface plasmon resonance
SREBP1c	sterol regulatory element-binding protein 1c
STC-1	enteroendocrine cell line
STZ	streptozotocin
T2D	type 2 diabetes
TBIL	total bilirubin
TG	triglycerides
TGF-β/TGF-β1	transforming growth factor beta/beta
TLC/HPLC	thin-layer chromatography/high-performance liquid chromatography
TNF-α	tumor necrosis factor alpha
TROY/TNFRSF19	tumor necrosis factor receptor superfamily member 19
TUNEL	terminal deoxynucleotidyl transferase dUTP nick-end labeling
UHPLC-MS/MS	ultra-high-performance liquid chromatography–tandem mass spectrometry
UPR	unfolded protein response
VC	vascular complications
VLDL	very-low-density lipoprotein
VE-cadherin	vascular endothelial cadherin
VEGF-A	vascular endothelial growth factor A
VEGFR2	vascular endothelial growth factor receptor 2
WB	Western blot
WT	wild type
XBP1s	spliced X-box binding protein 1
ZO-1	zonula occludens 1

## References

[1] International Diabetes Federation (2025). IDF Diabetes Atlas.

[2] Suneja S., Gangopadhyay S., Saini V., Dawar R., Kaur C. (2021). Emerging Diabetic Novel Biomarkers of the 21st Century. Ann. Natl. Acad. Med. Sci..

[3] International Diabetes Federation (2017). IDF Diabetes Atlas.

[4] Ahluwalia T.S., Kilpeläinen T.O., Singh S., Rossing P. (2019). Editorial: Novel Biomarkers for Type 2 Diabetes. Front. Endocrinol..

[5] Zheng Y., Ley S.H., Hu F.B. (2018). Global Aetiology and Epidemiology of Type 2 Diabetes Mellitus and Its Complications. Nat. Rev. Endocrinol..

[6] Makhdoomi M.J., Chander G., Showkat W., Golhani V., Sheikh A.A. (2023). Biomarkers in Diabetes Mellitus (DM)—With a Special Focus on miRNAs as Future Markers for Diagnosis of DM. Biointerface Res. Appl. Chem..

[7] Galicia-Garcia U., Benito-Vicente A., Jebari S., Larrea-Sebal A., Siddiqi H., Uribe K.B., Ostolaza H., Martín C. (2020). Pathophysiology of Type 2 Diabetes Mellitus. Int. J. Mol. Sci..

[8] Fuchsberger C., Flannick J., Teslovich T.M., Mahajan A., Agarwala V., Gaulton K.J., Ma C., Fontanillas P., Moutsianas L., McCarthy D.J. (2016). The Genetic Architecture of Type 2 Diabetes. Nature.

[9] Dimas A.S., Lagou V., Barker A., Knowles J.W., Mägi R., Hivert M.-F., Benazzo A., Rybin D., Jackson A.U., Stringham H.M. (2014). Impact of Type 2 Diabetes Susceptibility Variants on Quantitative Glycemic Traits Reveals Mechanistic Heterogeneity. Diabetes.

[10] Schellenberg E.S., Dryden D.M., Vandermeer B., Ha C., Korownyk C. (2013). Lifestyle Interventions for Patients With and at Risk for Type 2 Diabetes: A Systematic Review and Meta-Analysis. Ann. Intern. Med..

[11] Franks P.W., Pearson E., Florez J.C. (2013). Gene-Environment and Gene-Treatment Interactions in Type 2 Diabetes. Diabetes Care.

[12] Bellou V., Belbasis L., Tzoulaki I., Evangelou E. (2018). Risk Factors for Type 2 Diabetes Mellitus: An Exposure-Wide Umbrella Review of Meta-Analyses. PLoS ONE.

[13] Stumvoll M., Goldstein B.J., Van Haeften T.W. (2005). Type 2 Diabetes: Principles of Pathogenesis and Therapy. Lancet.

[14] Rieusset J., Bouzakri K., Chevillotte E., Ricard N., Jacquet D., Bastard J.-P., Laville M., Vidal H. (2004). Suppressor of Cytokine Signaling 3 Expression and Insulin Resistance in Skeletal Muscle of Obese and Type 2 Diabetic Patients. Diabetes.

[15] Guo H., Wu H., Li Z. (2023). The Pathogenesis of Diabetes. Int. J. Mol. Sci..

[16] Cerf M.E. (2013). Beta Cell Dysfunction and Insulin Resistance. Front. Endocrinol..

[17] Fu Z., Gilbert E.R., Liu D. (2013). Regulation of Insulin Synthesis and Secretion and Pancreatic Beta-Cell Dysfunction in Diabetes. Curr. Diabetes Rev..

[18] Christensen A.A., Gannon M. (2019). The Beta Cell in Type 2 Diabetes. Curr. Diab Rep..

[19] Halban P.A., Polonsky K.S., Bowden D.W., Hawkins M.A., Ling C., Mather K.J., Powers A.C., Rhodes C.J., Sussel L., Weir G.C. (2014). β-Cell Failure in Type 2 Diabetes: Postulated Mechanisms and Prospects for Prevention and Treatment. Diabetes Care.

[20] Van Schaftingen E., Gerin I. (2002). The Glucose-6-Phosphatase System. Biochem. J..

[21] Oh K.-J., Han H.-S., Kim M.-J., Koo S.-H. (2013). CREB and FoxO1: Two Transcription Factors for the Regulation of Hepatic Gluconeogenesis. BMB Rep..

[22] Montal E.D., Dewi R., Bhalla K., Ou L., Hwang B.J., Ropell A.E., Gordon C., Liu W.-J., DeBerardinis R.J., Sudderth J. (2015). PEPCK Coordinates the Regulation of Central Carbon Metabolism to Promote Cancer Cell Growth. Mol. Cell.

[23] Leclercq I.A., Da Silva Morais A., Schroyen B., Van Hul N., Geerts A. (2007). Insulin Resistance in Hepatocytes and Sinusoidal Liver Cells: Mechanisms and Consequences. J. Hepatol..

[24] Samuel V.T., Shulman G.I. (2012). Mechanisms for Insulin Resistance: Common Threads and Missing Links. Cell.

[25] Hotamisligil G.S. (2010). Endoplasmic Reticulum Stress and the Inflammatory Basis of Metabolic Disease. Cell.

[26] Nauck M.A., Müller T.D. (2023). Incretin Hormones and Type 2 Diabetes. Diabetologia.

[27] Gautier J.-F., Choukem S.-P., Girard J. (2008). Physiology of Incretins (GIP and GLP-1) and Abnormalities in Type 2 Diabetes. Diabetes Metab..

[28] Dietrich M.O., Horvath T.L. (2013). Hypothalamic Control of Energy Balance: Insights into the Role of Synaptic Plasticity. Trends Neurosci..

[29] Morton G.J., Cummings D.E., Baskin D.G., Barsh G.S., Schwartz M.W. (2006). Central Nervous System Control of Food Intake and Body Weight. Nature.

[30] Schwartz M.W., Woods S.C., Porte D., Seeley R.J., Baskin D.G. (2000). Central Nervous System Control of Food Intake. Nature.

[31] Wang X., Yang Y., Zhao D., Zhang S., Chen Y., Chen Y., Feng K., Li X., Han J., Iwakiri Y. (2022). Inhibition of High-Fat Diet–Induced Obesity via Reduction of ER-Resident Protein Nogo Occurs through Multiple Mechanisms. J. Biol. Chem..

[32] Fournier A.E., GrandPre T., Strittmatter S.M. (2001). Identification of a Receptor Mediating Nogo-66 Inhibition of Axonal Regeneration. Nature.

[33] Slieker R.C., Donnelly L.A., Akalestou E., Lopez-Noriega L., Melhem R., Güneş A., Abou Azar F., Efanov A., Georgiadou E., Muniangi-Muhitu H. (2023). Identification of Biomarkers for Glycaemic Deterioration in Type 2 Diabetes. Nat. Commun..

[34] Slieker R.C., Donnelly L.A., Lopez-Noriega L., Muniangi-Muhitu H., Akalestou E., Sheikh M., Georgiadou E., Giordano G.N., Åkerlund M., Ahlqvist E. (2021). Novel Biomarkers for Glycaemic Deterioration in Type 2 Diabetes: An IMI RHAPSODY Study. medRxiv.

[35] Gong K., Xue C., Feng Z., Pan R., Wang M., Chen S., Chen Y., Guan Y., Dai L., Zhang S. (2024). Intestinal Nogo-B Reduces GLP1 Levels by Binding to Proglucagon on the Endoplasmic Reticulum to Inhibit PCSK1 Cleavage. Nat. Commun..

[36] Schwab M.E. (2010). Functions of Nogo Proteins and Their Receptors in the Nervous System. Nat. Rev. Neurosci..

[37] Roy A., Pathak Z., Kumar H. (2021). Strategies to Neutralize RhoA/ROCK Pathway after Spinal Cord Injury. Exp. Neurol..

[38] Wang T., Xiong J.-Q., Ren X.-B., Sun W. (2012). The Role of Nogo-A in Neuroregeneration: A Review. Brain Res. Bull..

[39] Wang X., Chun S.-J., Treloar H., Vartanian T., Greer C.A., Strittmatter S.M. (2002). Localization of Nogo-A and Nogo-66 Receptor Proteins at Sites of Axon–Myelin and Synaptic Contact. J. Neurosci..

[40] Lang B.T., Wang J., Filous A.R., Au N.P.B., Ma C.H.E., Shen Y. (2014). Pleiotropic Molecules in Axon Regeneration and Neuroinflammation. Exp. Neurol..

[41] Acevedo L., Yu J., Erdjument-Bromage H., Miao R.Q., Kim J.-E., Fulton D., Tempst P., Strittmatter S.M., Sessa W.C. (2004). A New Role for Nogo as a Regulator of Vascular Remodeling. Nat. Med..

[42] Miao R.Q., Gao Y., Harrison K.D., Prendergast J., Acevedo L.M., Yu J., Hu F., Strittmatter S.M., Sessa W.C. (2006). Identification of a Receptor Necessary for Nogo-B Stimulated Chemotaxis and Morphogenesis of Endothelial Cells. Proc. Natl. Acad. Sci. USA.

[43] Bonal C.B., Baronnier D.E., Pot C., Benkhoucha M., Schwab M.E., Lalive P.H., Herrera P.L. (2013). Nogo-A Downregulation Improves Insulin Secretion in Mice. Diabetes.

[44] Chen Y., Hu W., Li Q., Zhao S., Zhao D., Zhang S., Wei Z., Yang X., Chen Y., Li X. (2021). NGBR Is Required to Ameliorate Type 2 Diabetes in Mice by Enhancing Insulin Sensitivity. J. Biol. Chem..

[45] Geoffroy C.C., Zheng B. (2015). Myelin-Associated Inhibitors in Axonal Growth after Central Nervous System Injury. Neural Regeneration.

[46] Relton J.K., Weinreb P.H. (2008). STRATEGIES TO INHIBIT SIGNALING THROUGH NOGO RECEPTOR 1 FOR SPINAL CORD INJURY AND STROKE. CNS Regeneration.

[47] Fischer D., Leibinger M. (2012). Promoting Optic Nerve Regeneration. Prog. Retin. Eye Res..

[48] Dave B.P., Shah K.C., Shah M.B., Chorawala M.R., Patel V.N., Shah P.A., Shah G.B., Dhameliya T.M. (2023). Unveiling the Modulation of Nogo Receptor in Neuroregeneration and Plasticity: Novel Aspects and Future Horizon in a New Frontier. Biochem. Pharmacol..

[49] Hetz C. (2012). The Unfolded Protein Response: Controlling Cell Fate Decisions under ER Stress and Beyond. Nat. Rev. Mol. Cell Biol..

[50] Christianson J.C., Ye Y. (2014). Cleaning up in the Endoplasmic Reticulum: Ubiquitin in Charge. Nat. Struct. Mol. Biol..

[51] Rorsman P., Renström E. (2003). Insulin Granule Dynamics in Pancreatic Beta Cells. Diabetologia.

[52] Sung B.-J., Lim S.-B., Yang W.-M., Kim J.H., Kulkarni R.N., Kim Y.-B., Lee M.-K. (2022). ROCK1 Regulates Insulin Secretion from β-Cells. Mol. Metab..

[53] Hammar E., Tomas A., Bosco D., Halban P.A. (2009). Role of the Rho-ROCK (Rho-Associated Kinase) Signaling Pathway in the Regulation of Pancreatic β-Cell Function. Endocrinology.

[54] Krell S., Hamburg A., Gover O., Molakandov K., Leibowitz G., Sharabi K., Walker M.D., Helman A. (2025). Beta Cells Intrinsically Sense and Limit Their Secretory Activity via mTORC1-RhoA Signaling. Cell Rep..

[55] Park E.J., Grabińska K.A., Guan Z., Stránecký V., Hartmannová H., Hodaňová K., Barešová V., Sovová J., Jozsef L., Ondrušková N. (2014). Mutation of Nogo-B Receptor, a Subunit of Cis-Prenyltransferase, Causes a Congenital Disorder of Glycosylation. Cell Metab..

[56] Edani B.H., Grabińska K.A., Zhang R., Park E.J., Siciliano B., Surmacz L., Ha Y., Sessa W.C. (2020). Structural Elucidation of the *Cis* -Prenyltransferase NgBR/DHDDS Complex Reveals Insights in Regulation of Protein Glycosylation. Proc. Natl. Acad. Sci. USA.

[57] Liu X., Zuo Z., Liu W., Wang Z., Hou Y., Fu Y., Han Y. (2014). Upregulation of Nogo Receptor Expression Induces Apoptosis of Retinal Ganglion Cells in Diabetic Rats. Neural Regen. Res..

[58] Yang Q., Zhang C., Xie H., Tang L., Liu D., Qiu Q., Luo D., Liu K., Xu J.-Y., Tian H. (2021). Silencing Nogo-B Improves the Integrity of Blood-Retinal Barrier in Diabetic Retinopathy via Regulating Src, PI3K/Akt and ERK Pathways. Biochem. Biophys. Res. Commun..

[59] Irakoze L., Ma L., Gu Y., Chen X., Zeng F., Luo R., Lai Y., Li X., Chen S., Banderembako P. (2026). Levels and Effects of Nogo-B in Patients With Type 2 Diabetes or Hyperglycemic HUVEC Model. Endocrinol. Diabetes Metab..

[60] Hernandez-Diaz I., Pan J., Ricciardi C.A., Bai X., Ke J., White K.E., Flaquer M., Fouli G.E., Argunhan F., Hayward A.E. (2019). Overexpression of Circulating Soluble Nogo-B Improves Diabetic Kidney Disease by Protecting the Vasculature. Diabetes.

